# Multi Level Approach for Segmentation of Interstitial Lung Disease (ILD) Patterns Classification Based on Superpixel Processing and Fusion of *K*-Means Clusters: SPFKMC

**DOI:** 10.1155/2022/4431817

**Published:** 2022-10-22

**Authors:** Anni U. Gupta, Sarita Singh Bhadauria

**Affiliations:** ^1^E&TC, UIT-RGPV, Bhopal, India; ^2^School of Information Technology, RGPV, Bhopal, India

## Abstract

During the COVID-19 pandemic, huge interstitial lung disease (ILD) lung images have been captured. It is high time to develop the efficient segmentation techniques utilized to separate the anatomical structures and ILD patterns for disease and infection level identification. The effectiveness of disease classification directly depends on the accuracy of initial stages like preprocessing and segmentation. This paper proposed a hybrid segmentation algorithm designed for ILD images by taking advantage of superpixel and *K*-means clustering approaches. Segmented superpixel images adapt the better irregular local and spatial neighborhoods that are helpful to improving the performance of *K*-means clustering-based ILD image segmentation. To overcome the limitations of multiclass belongings, semiadaptive wavelet-based fusion is applied over selected *K*-means clusters. The performance of the proposed SPFKMC was compared with that of 3-class Fuzzy *C*-Means clustering (FCM) and *K*-Means clustering in terms of accuracy, Jaccard similarity index, and Dice similarity coefficient. The SPFKMC algorithm gives an accuracy of 99.28%, DSC 98.72%, and JSI 97.87%. The proposed Fused Clustering gives better results as compared to traditional K-means clustering segmentation with wavelet-based fused cluster results.

## 1. Introduction

Segmentation of the image is a critical step for detecting early lung anomalies, diagnosis, and planning of therapy. The purpose of image segmentation is to separate nonconnected clusters of the image regions based on feature homogeneity, viz. color intensity, shape, texture, etc. Early diagnosis of disease encourages early treatment, which improves the opportunity for patient endurance. But during the pandemic of COVID-19, resource limitation has significantly delayed the routine diagnosis of lung patients. The nature of lung infections also has a great deal of interest and is highly required to be analyzed. Lung segmentation analysis and classification [1] can significantly help in the early diagnosis of disease patterns. The increasing threat of lung diseases like COVID is the major motivation for designing an efficient segmentation algorithm for higher level analysis or classification. Another motivation behind interstitial lung disease (ILD) research is the low survival time of an average 5 years for ILD patients. This shows the seriousness of designing the early diagnosis algorithm for these images.

ILD is a collection of diseases that induce progressive scarring of lung tissue. It is extremely common in India and worldwide, and it has a higher risk of mortality if not detected at early stages [1–3]. ILD is a case of chronic obstructive pulmonary disease (COPD), and is usually an umbrella term encompassing both chronic bronchitis and emphysema. The diagnosis of ILD diseases is a challenging task at hand. Clustering-based segmentation algorithms [4, 5] are frequently adopted to differentiate clusters belonging to the same classes. A huge number of segmentation methodologies have been designed in the recent past. But still, there are certain issues to be addressed, taking ILD images into consideration.

**Figure 1:**
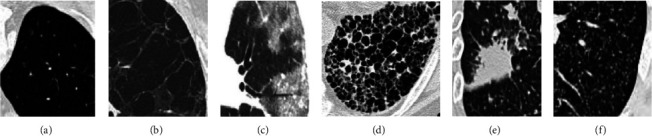
Different ILD patterns such as (a) healthy (b) emphysema (c) ground glass (d) fibrosis (e) consolidation (f) micro-nodules.

High-resolution CT (HRCT) [1] imaging is the standard way of observing the patterns of lung alterations specifically in ILD images. There are various ILD patterns observed typically in HRCT images viz. Emphysema, healthy, fibrosis, ground glass, consolidation, and micro-nodules. Examples of these tissue patterns are shown in Figure 1. Among all fibrosis and consolidation, the serious and frequently found ILD cases need early detection.

The typical flow for the detection of ILD images consists of preprocessing, segmentation, feature extraction, and classification stages. The objectives of preprocessing methods are intensity improvement, noise removal, and enhancement of an image. Segmentation is used for the extraction of features from the desired ROI (Region of Interest). The segmented ROI determined by lung segmentation is utilized to find infectious objects and boundaries within ILD images. Therefore, medical image segmentation assumes a huge job in clinical diagnosis. Many image segmentation approaches [6–9] have been proposed in the past. There are certain algorithms which use segmented features for ILD pattern classifications. The classification of ILD patterns is a tough and complicated process. Most existing classification methods are a bit time consuming due to the use of supervised learning models. It is an open challenge to design a simple and easy way of efficient pattern classification by adopting unsupervised techniques. Therefore, first, this paper aims to design an efficient segmentation approach by eliminating identified issues. Then the simple and efficient correlation matching and parametric-based classification are presented using the segmented results.

### 1.1. Classification of Segmentation Algorithms

**Figure 2:**
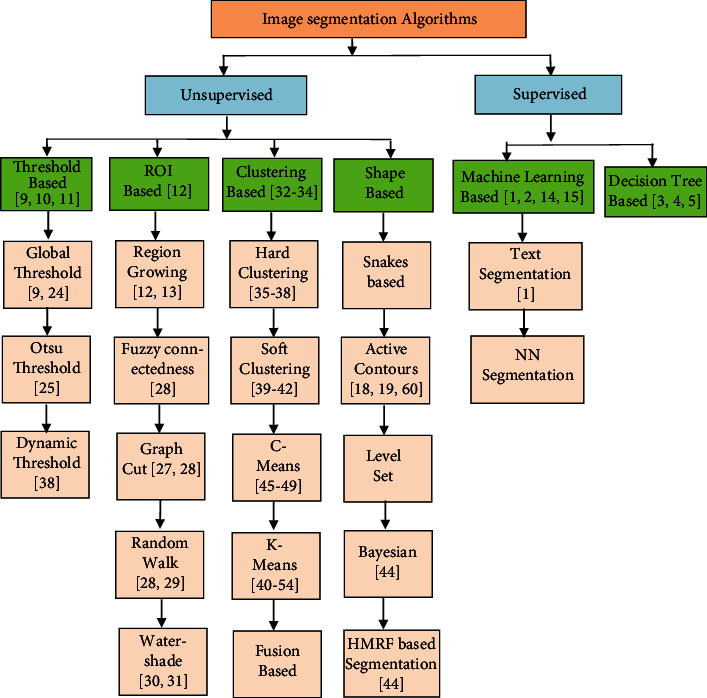
Classification Tree of image segmentation algorithms.

Broadly, image segmentation algorithms are classified into classes of supervised and unsupervised algorithms as shown in Figure 2. The segmentation of medical images is mostly implemented as an unsupervised problem. Our major concern in the paper is to increase the performance of clustering-based segmentation algorithms. Clustering is the process of segmenting patterns of the objects such that members of the same cluster are more correlated to each other, compared to members belonging to other clusters. Fuzzy-based clustering is a common and popular approach in literature. Fuzzy *C* - Means Clustering (FCM) is the most popular algorithm among the fuzzy clustering methods. The method is used for segmenting the images since it offers robustness to ambiguities and thus retains better information than conventional algorithms work over noise-less imaging environments. The nomenclature used for the study is described in Table 1.

The remaining paper is sequentially organized as follows: the next section gives a brief overview of several existing segmentation and classification systems an ILD brief summary of literature is provided relevant to current work. Section 3 addressed various design challenges for ILD image segmentation. The proposed design frameworks are sequentially discussed in the modified system developed step by step in section 4; the results and discussion of segmentation and classification are presented in section 5. Futhermore, a comprehensive parametric performance-based comparison is provided, followed by the conclusions in the next section.

## 2. Background and Related Works

In the literature, there are many types of segmentation algorithms applied to medical images, such as thresholding [10, 11], region growing [12, 13], machine learning [14, 15], deep-learning [16, 17], active contour [18, 19], quantum-inspired computing [20, 21], and computational intelligence [22, 23]. Therefore, this section sequentially and individually reviews the related recent developments in unsupervised and supervised categories.

### 2.1. Review of Supervised Segmentation

There are various lung segmentation methods designed in recent times using supervised machine learning [14, 15] techniques using texture features and a deep learning approach for segmentation of lung images [1]. But the method is computationally complex and needs accurate training data. The efficiency of segmentation and classification in supervised learning methods depends on the degree of closeness between quarry and training images. Usually, machine learning and decision tree-based segmentation approaches come under the category of supervised segmentation methodologies.

#### 2.1.1. Machine Learning Based Segmentations

Researcher in [2] has introduced a deep learning algorithm for COVID-19-inspired lung image segmentation extended to [16, 17]. They employed SegNet and the *U*-Net network to demonstrate deep learning-based infected tissue segmentation in lung images. However, learning-based approaches have a key drawback in that they require the training of image segmentation characteristics. Self-supervised autonomous lung image segmentation over the COVID-19 database has been described in [3], notably for age-related illnesses. For a deep learning system to be effective, the data sample size must be large. As a result, learning without supervision may be preferred, but research into this area is still ongoing. The authors in [4] described the review of different clustering and learning-based image segmentation methods. Among the different segmentation techniques, the Convolution Neural Network (CNN) is used in [5] for lung image classification and segmentation. The method was specifically designed for the of lung images. As [7] have presented the statistical classification approach for lung images.

#### 2.1.2. Decision Tree Based Segmentation

Textural features were used to create decision trees, which were subsequently used in the previously suggested method, according to [7]. The use of decision trees to select new informative signs resulted in higher accuracy. In [8], they have given excellent research on decision tree-guided segmentation for use in land coverage classification. However, it appears that the procedure is a little difficult to compute. Another drawback of learning algorithms is that their structures are susceptible to minor changes in data properties. As a result, not ideal for lung segmentation. As a result, it is deep learning-based methods have recently been chosen over decision tree approaches.

### 2.2. Review of Un-Supervised Segmentation

The simplest of segmentation algorithms is to use global or variable thresholding-based [10, 11] methods [5, 6] these methods present the global segmentation based on the threshold selection approach. Researchers in [24] have performed segmentation based on thresholding over infrared thermal images. Their thresholding algorithm reduces the computational cost by locating the optimal threshold values. An approach described by the improved region of interest (I-ROI) segmentation technique for satellite images also enhances the contrast of the region by [25]. Proposed a new segmentation method [26] based on the conditional region growing [12, 13] approach for mammographic images, but the method is dependent on the order of the pixels processed thus results may vary for medical images. Introduce improved region growing (IRG) method [27] to segment lung tumor with less time and more accuracy. In the [28] proposed a method of segmenting medical images based on neural networks (NN) and fuzzy connectedness. But, the performance of the fuzzy method is expected to vary with image databases. While in [29, 30], they presented different approaches to global and local segmentation methods for the underwater images. These methods compare the pixel brightnesses with a selected threshold to segment the images.

The graph cuts [31], random walks [32], watershed transform [33], clustering [34, 35], active contour model [18, 19, 36], hidden Markov random field (HMRF) [37], fuzzy entropy [38], etc. are included in unsupervised image segmentation and are mostly popular and useful for their simplicity without depending on training samples and labels. Machine learning-based methods are among the supervised approaches. Although certain supervised systems, such as artificial neural network (ANN) [39], Convolution neural network (CNN) [40], and fully convolution networks (FCNs) [41, 42], can achieve picture segmentation by applying feature learning, they require a significant amount of testing and labeling. In addition, because CNN and FCN essentially perform picture categorization, the segmentation output has a coarse contour.

The focus of this research is on unsupervised image segmentation. Clustering is one of the most important and widely used unsupervised methods for grayscale and color image segmentation since it is based on partitioning into homogeneous clusters, making it more effective for real-world issues. The focus of this paper is on image segmentation based on clustering. Clustering-based techniques are those that divide an image's approach into clusters of pixels with similar characteristics. Data clustering is a method of dividing information elements into clusters such that elements in comparable clusters are more connected to each other than elements in other clusters.

### 2.3. Clustering Based Approaches

Accurate segmentation is nearly impossible in real life due to the presence of noise. There are two basic types of clustering hard clustering (HC) and soft clustering (SC) [43]. Some of the soft clustering techniques are FCM (fuzzy c-means) [44] which is based on grouping on an objective function, which is the most widely used and popular technique, FCS (fuzzy c-shells) [45] and FLAME [46] which is used for large dataset; hard clustering categories such as *K*-means, which is low time complexity.

Soft clustering approaches [44] include FCM, which is a more natural type of grouping. As a corollary, soft clustering techniques are ideal for image segmentation where strict division is not required. *K*-means clustering, as proposed [47], is included in Hard Clustering (HC). The high-accuracy adaptive *K*-means segmentation technique is a simple clustering approach that divides an image into a collection of clusters, with every pixel belonging to just one cluster. The disadvantage is that this method takes longer to execute. In other words, each pixel can only be assigned to one cluster. Membership functions containing values of 1 or 0 are used in these approaches, meaning that a pixel may belong to a given cluster or not.

There are many approaches being proposed using the FCM and the *K*-mean-based clustering techniques as in [48–51]. *K*-mean clustering is widely used for medical image segmentation purposes. However, both *K*-means and FCM are vulnerable to noise because picture segmentation ignores the local spatial information of pixels. Clustering segmentation approaches frequently overlook spatial information, resulting in poor image segmentation results. In addition, the desired clusters may belong to multiple classes as *k* increases. And adoption of suitable cluster numbers is an open challenge for *k* mean cases. Many times wrong selection of clusters may lead to an empty cluster problem, although this issue can be resolved by reexecuting the algorithm.

### 2.4. Fuzzy Super Pixel Approaches

On the other hand, superpixel can reduce the myriad of separate pixels in an image by replacing every pixel in a segment with the superpixel region's mean value [52, 53]. Recently, we demonstrated a random forest (RF) and deep convolutional network-based segmentation method and have used multi-scale superpixels features for pathological lung CT image segmentation [54]. They evaluated performance based on statistical similarity indexes as parameters. Various researchers, on the other hand, have attempted to develop a strategy for accurately detecting critical ILD. Therefore, this session presented the different segmentation approaches utilized for ILD images. In this study, superpixel technology is used to capture adaptive local spatial information at early stages of an algorithm. The use of traditional imaging tools to detect ILD is limited due to the fuzzy boundary and tiny size of the infected zone. FCM-based hybrid segmentation using the region-growing approach [55], but this approach may have a dependency on pixel selection. The impact of the motion artefacts on the HRCT images of the lungs [56]. In the [57], researchers presented a good review of the various approaches to ILD image pattern classification.

For segmentation, thFCM method is utilized [58], which takes fewer iterations to reach the global best solution however, failing to segment images damaged by noise, the approach was demonstrated in the underwater imagery case. The diseases can be easily segmented with improved precision using the registration approach [59]. However, the highest border placement error may occur, and the technology can only be applied to clinical practices after extensive testing with numerous scans. Because of its shorter execution time and better accuracy, the model-based method was found to operate effectively in the presence of a diseased lung. A form model termed active contours or snakes [60], which are self-adapting and autonomous in their search for the smallest amount of energy, and may be used to follow dynamic objects in both temporal and spatial dimensions. Snakes, on the other hand, are prone to becoming caught in local minimum states, which necessitates a longer computation time. The *K*-mean clustering technique is presented based on FCM [61, 62], which uses segmentation of the lung cancer images. But it develops anomalies and has probabilistic performance in segmentation. The unsupervised Bayesian segmentation approach was proposed [63]. The supervised FCN [64] base classification and segmentation approach is good, but the method needs good trading data knowledge.

Although many better methods handle the problem by incorporating local geographic data into the objective function, this increases computational complexity. Fortunately, superpixel [65, 66] can fix the problem. Superpixel is an image preprocessing program that oversegments images into several small pieces. As [67] demonstrated, an entropy rate maximization based random walk and a graph cut-dependent superpixel segmentation technique were introduced.

In an image, a superpixel zone is often defined as perceptually uniform and homogeneous patches [67]. For two reasons,superpixel can improve the efficacy and efficiency of image segmentation. On the one hand, Superpixel can pre-segment images using local spatial information. The presegmentation enhances the spatial data accessible in the immediate vicinity. In [68] have presented the linear spectral clustering-based image segmentation using the superpixel based approach. Some other segmentation methods are recently given in [69–71]. The wavelet-based fusion strategy has been presented [72] for increasing the quality of medical images. Their fusion method is suitable enough to be used in the current paper for cluster fusion. The summary of different ILD image segmentation and classification methods are given in Table 2.

## 3. Challenges of ILD Image Segmentation and Classification

**Figure 3:**
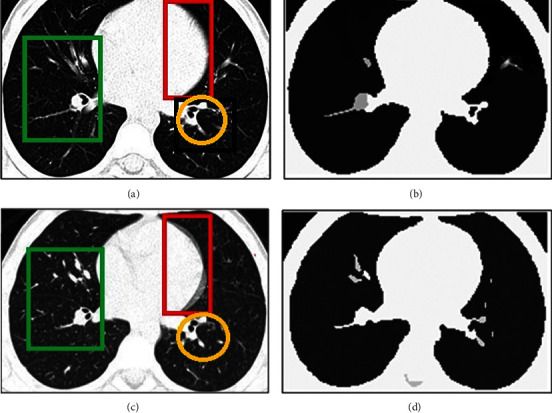
An impact of motion artefact on the segmentation performance for volumetric HRCT images with and without motion artefacts [6]. (a) Original VHRCT image, (b) Segmented original with superpixel. (c) Motion artifact blured VHRCT, (d) Segmented VHRCT with superpixel.

**Figure 4:**
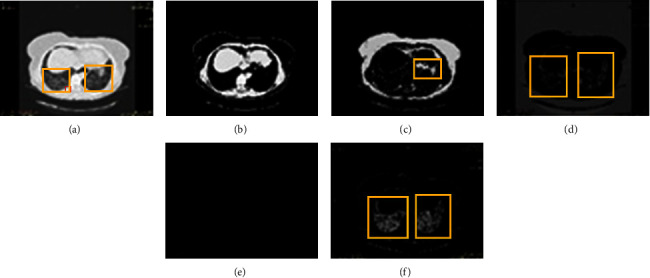
An example of the multi-cluster belongings of the objects of interest for ILD images. (a) Original image, (b) *K*-mean cluster 1, (c) *K*-mean cluster 2, (d) *K*-mean cluster 3, (e) *K*-mean cluster 4, (f) *K*-mean cluster 5.

Identification of ILD diseases is primarily based on the extraction of features using the segmentation of images. In spite of existing methods, it is still an open challenge to improve ILD segmentation efficiency. Since during the COVID-19 there is lot of changes in lungs patterns and identification is must. These challenging ILD segmentation issues have been sequentially described in this section:

- Acquisition Artefacts: There are many artefacts [6] present in the ILD image during the image capturing time. These artrfacts may be due to the hardening of metallic beams, unintentional patient motions, or variably of ILD image acquisition methodologies. These artifacts make it difficult to accurately segment and analyze ILD lung patters. Many times, due to human motion, the captured HRCT images suffer from artifacts and blurriness [6]. Examples of motion artifact impact over segmentation quality are represented in Figure 3. The motion artifacts impacts are respectively represented by red, green, and orange boxes.
- It can be seen that the presence of the artifacts can significantly change the segmented content. Thus, it is mandatory to avoid the artifacts at the time of acquisition only.
- Preprocessing: The efficiency of preprocessing approaches can directly impact on ILD classification. The preprocessing inaccuracy may lead to inaccurate segmentation of desired ILD patterns and in turn reproduce the inaccurate classification. The preprocessing stages can also be used for improving the contrast or for separation of the background of the ILD images. Sometimes, excessive contrast enhancement can produce the doubling effects in ILD segmented images.
- Aging Impacts: The heterogeneity varies due to different age groups or pulmonary structures. Therefore, the efficiency of the segmentation methods varies with different lung imaging datasets. Since aging significantly varies the ILD image patterns.
- Structural Heterogeneity: The segmentation of the lungs is a very challenging problem due to the homogeneities in the lung region, pulmonary structures of similar densities such as arteries, veins, bronchi, and bronchioles.
- Speed and uncertainty of clustering. There were many FCM-based segmentation approaches designed for performance improvement. The major challenge of clustering-based segmentation is the longer execution time, and due to the breaking up of local spatial features, may lead to uncertain segmented results. It means that, with the different local features of a database, the performance of FCM-based segmentations is uncertain.
- Multi Cluster Belongings: The C-Mean and the K-Mean clustering are the most widely used methods for segmentation. It is observed that the selection of the number of clusters for K-mean clustering is an open field of discussion. Since there is a significant probability that desired objects will belong to two or more clusters. Thus, selecting an optimum cluster is a challenging task. Many times, the clustered output does not contain the complete desired region. An example of multi-cluster belonging is shown in Figure 4. The desired object of interest is shown by the rectangle boxes showing the multi-cluster case. Desired features are distributed over clusters.

In this paper, all these challenges except the acquisition part are considered, and a systematic algorithm is designed to overcome the problems. The hybrid segmentation methodology is presented for the feature extraction and then the 2D correlation-based image classification/matching is presented as a case study on ILD classification.

## 4. Proposed Hybrid Fusion Based Segmentation

**Figure 5:**
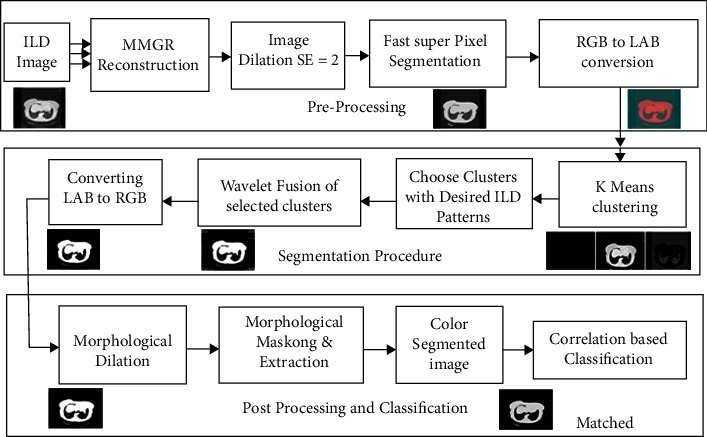
Block diagram of the proposed nethodology in three stage process.

Paper proposed to design the efficient hybrid *K*-mean clustering approach with wavelet-based cluster fusion for efficiency improvement; the proposed method is a three-phase approach as preprocessing, segmentation and postanalysis and classification as shown in the three rows diagram in Figure 5. In the first pre-processing stage, the paper proposed using the existing superpixel segmentation concept for background separation to obtain better local spatial information use, improving the quality of the clustering-based segmentation region. It is suggested to convert the RGB image to lab color space before segmentation. In the second phase, *K*-means clustering approach is presented over a superpixel segmented image. Since *K*-means clustering depends on the global feature, the combination of superpixel and *K*-means clustering is able to improve image segmentation results.

The paper computes a modified clustering algorithm based on the obtained superpixel image. But it is observed that local desired features may belong to multiple clusters. Thus, to improve the efficiency in the third phase, desired clusters are fused to improve the segmentation quality. Then morphological processing is used to reproduce the color segmentation of the desired image features.

### 4.1. Super Pixel Calculation

In the preprocessing for background separation, the paper uses Watershed to offer superpixel images using multi-scale morphological-based restoration. To address the problem, computational calculations based on changing the gradient image of the original image have been presented. Multi-scale morphological basis gradients reconstruction (MMGR) is a simple and effective approach for overcoming oversegmentation because it is able to retain object contour information while reducing noise and unnecessary gradient features. The following is an explanation of what morphological reproduction details

(1) $$\begin{array}{c} \left\{\begin{array}{c} M_{f}^{Ɛ}\left(g\right)={Ɛ}_{\left(f\right)}^{\left(i\right)}\left(g\right)\text{,}\\ M_{f}^{\delta }\left(g\right)=\delta_{f}^{\left(i\right)}\left(g\right)\text{,} \end{array}\right. \end{array}$$

where *M*^*Ɛ*^ and *M*^*δ*^ respectively, stand for the morphological erosion and dilation for reconstruction, and *f* stands for an original input image; i.e. the mask-based image, *g* stands for the marker image, and is the dilation operation. Since morphological-based erosion and dilation are multiple operators, they usually appear in pairs, such as morphological basis opening-closing operators. Although these operators have a more robust capability for extracting features or, on the other extreme, noise elimination, morphological opening and closing are more well-known than erosion and dilation. As a result, the morphological opening and closing-based reconstructions, represented by *M*^*O*^ and *M*^*C*^, are distinguished as

(2) $$\begin{array}{c} \left\{\begin{array}{c} M^{O}\left(g\right)=R^{\delta }\left(R^{Ɛ}\right)\text{,}\\ M^{C}\left(g\right)=R^{Ɛ}\left(R^{\delta }\right)\text{,} \end{array}\right. \end{array}$$

where the marker image *g* is generally considered as.

*g* = *Ɛ*_*B*_ (*f*) in *M*^*δ*^ or *g* = *δ*_*B*_ (*f*) in *M*^*Ɛ*^.

*B* is a structuring element (SE). Both and are able to remove region minima in a gradient image to reduce over segmentation.

To adjust the quantity of districts in the superpixel image and contour precision, a reasonable SE is required; however, it is hard to pick an appropriate SE for various images. *M*^MC^ that is characterized as follows

(3) $$\begin{array}{c} M_{f}^{MC}\left(g,r_{1},r_{2}\right)=V\left\{M_{f}^{C}{\left(g\right)}_{Br1,}\;M_{f}^{C}\right.{\left(g\right)}_{Br+1\;},\ldots \ldots \ldots \left.M_{f}^{C}{\left(g\right)}_{Br2}\right\}\text{,} \end{array}$$

where *r*_1_ and *r*_2_ represent minima and maxima of *r*, *r*_1_ ≤ *r* ≤ *r*_2_.

**Figure 6:**
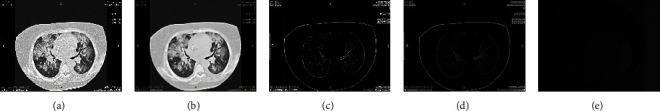
Super pixel image framework (a) original image (b) Gaussian filter image (c) Gradient Image (d) Morphological gradient reconstruction image (e) Watershed image.

By registering the point-wise maximum of these remade gradient images, a superb gradient image that eliminates the greater part of futile neighborhood minima while safeguarding significant edge details is obtained. Since a superpixel image conveys the spatial data of the image and diminishes the quantity of various colors, the superpixel image is better than an image quantized by clustering algorithms.  Figure 6, gives the framework of the superpixel image as below.

### 4.2. *K*-Means Clustering

One of the most widely used clustering methods is *K*-means clustering. The number of clusters is represented by *K*. This method's goal was to find the *K*-centroid for every cluster. Centroids were intelligently placed since a distinct location might yield a different outcome. Each point's data was linked to the closest centroid. When every point or pixel in the image had been considered, the iteration was finished. In each cycle, clustering center by centers were produced by remeasuring the known centroids. After obtaining *k*-new centroids, the data set point and the nearest new centroid needed to be assigned to their respective groups. The loop had been derived. The objective function is

(4) $$\begin{array}{c} J=\overset{k}{\underset{k-1}{\sum }}\overset{n}{\underset{i=1}{\sum }}{\left.{\left[X\right.}_{i}^{\left(j\right)}-{\left(C\right]2}_{j}\right]}^{2}\text{,} \end{array}$$

where, [*X*_*i*_^(*j*)^ − *C*_*j*_]^2^ = is selected Euclidian distance measure. *X*_*i*_^(*j*)^ = input data values. C_j_ = Center of the j^th^ cluster. *K*-means Algorithm

The clustering algorithm for the proposed *K*-mean segmentation is represented Algorithm 1.

**Figure 7:**
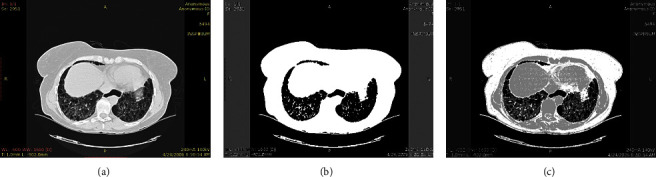
Comparison of the K-Mean clustering (a) Original image, (b) for *k* = 3 (c) for *k* = 5.

**Figure 8:**
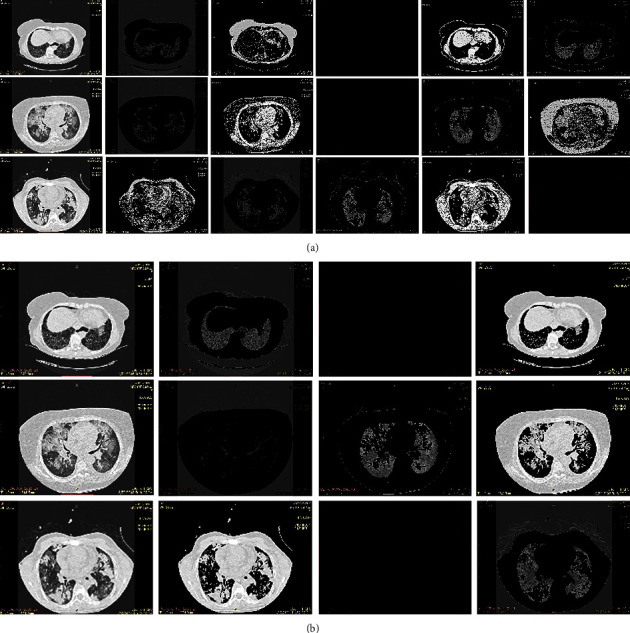
Resultant cluster Images for (a) Cluster 5 (b) Cluster 3.

Here we have to work with clusters 3 and 5 both. But the object details are in cluster 3 are more as compared to cluster 5, therefore cluster 3 selected.  Figure 7, shows the result with cluster 5 and cluster 3. And also Figure 8, shows the resultant cluster images with cluster 5 and cluster 3 along with the original images.

### 4.3. Wavelet Based Fusion

A wavelet-based picture fusion is used here. First, the wavelet transform is applied to images. Then, using a set of fusion rules, a fusion decision map is created. The fusion decision map can then be used to create a fused wavelet coefficient map to use the wavelet coefficients of the input images. Finally, the inverse wavelet transform is employed to create the fused image. Due to their multi-resolution properties, (DWT) based approaches have become popular; in this case, second level DWT was used. Pixel Level Maxima is the fusion method employed in this case (PLM). It's an image fusion technique based on intensity. We operate directly on the luminance of individual pixels in this method.

Let *A* (*x*, *y*) and *B* (*x*, *y*) are images to be fused. The decomposed low frequency sub images of *A* (*x*, *y*) and *B* (*x*, *y*) are *l*AJ (*x*, *y*) and *l*BJ (*x*, *y*). A decomposed high frequency subimages of *A* (*x*, *y*) and *B* (*x*, *y*) are *h*Aj, *k*(*x*, *y*) and *h*Bj, *k* (*x*, *y*). (*j* and *k* are the parameters of resolution, where *j* = 1,2,3,….*J* for every *j*, and *k* = 1,2,3,…, *k* for every *k*) There are different pixel level fusions methods like as: here, all the four subbands of the fused image *F* is simply formed by taking the wavelet coefficients from the source images that have the maximum value

(5) $$\begin{array}{c} F_{j,k}=\text{Mean}\left(A_{j,k}B_{j,k}\right)\text{,} \end{array}$$

where *l*A_j_(*x*, *y*) and *l*B_j_ (*x*, *y*) are low frequency subimages of *A* (*x,y*) and *B* (*x,y*).

**Figure 9:**
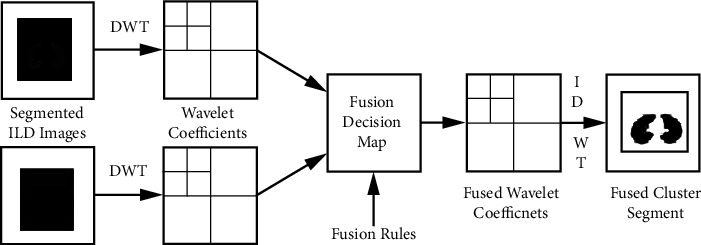
Wavelet based Image Fusion.

The algorithm performed for cluster fusion using wavelet-based fusion is represented in Algorithm 2.  Figure 9 shows the basic flow of wavelet-based image fusion.

**Figure 10:**
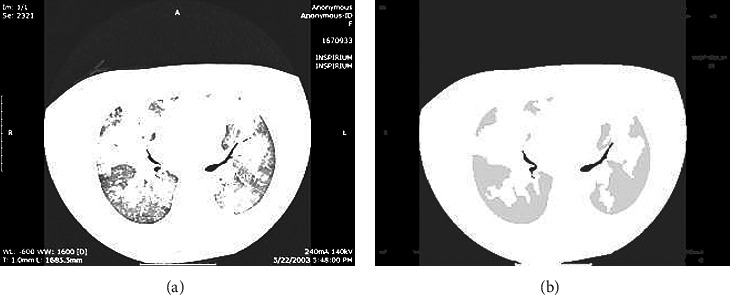
Resultant wavelet-based fused Images for (a) Original Image (b) Superpixel Image.

Here, the results obtained by *K*-means clustering consist of clusters up to 3. The images are fused into either cluster 1, 2 or cluster 2, 3 or cluster 1, 3 depending on the resultant images. These images are fused using wavelet-based fusion with the Daubechies4 (db4) wavelet, as in most cases this wavelet gives good results. As the objects in cluster 1 and cluster 2 are fused using wavelet-based fusion, the resultant fused images are as shown in Figure 10.

### 4.4. Morphological Image Processing

Morphological operations are used to remove imperfections introduced during segmentation. Numerical morphology is an apparatus of extricating image segments that is valuable in the representation and characterization of shape, such as, for example, boundaries, skeletons, and so forth.

Dilation Dilation is a process where the structuring element *B* is placed on the image *A* and it slides across the image, similar to a convolution. Steps involved in dilation:

1. On the off chance that the white pixel in the image harmonizes with the beginning of the organizing component, at that point there is no compelling reason to change; check the following pixel.
2. If the black pixel in the image coincides with the origin of the structuring element, then all the pixels covered by the structuring element are made black.

Notation: A⊕ B.

The structuring element can take any shape.

Erosion The erosion process is identical to dilation, but pixels are converted to “white” not “black.” Steps involved in erosion:

1. On the off chance that the “white” pixel in the image agrees with the cause of the organizing component, at that point there is no compelling reason to change; check the following pixel.
2. If a “black” pixel in an image coincides with the origin of a structuring element and at least one of the “black” pixels falls over a white pixel, then the “black” pixel, is converted to “white.” Notation: A Θ B

**Figure 11:**
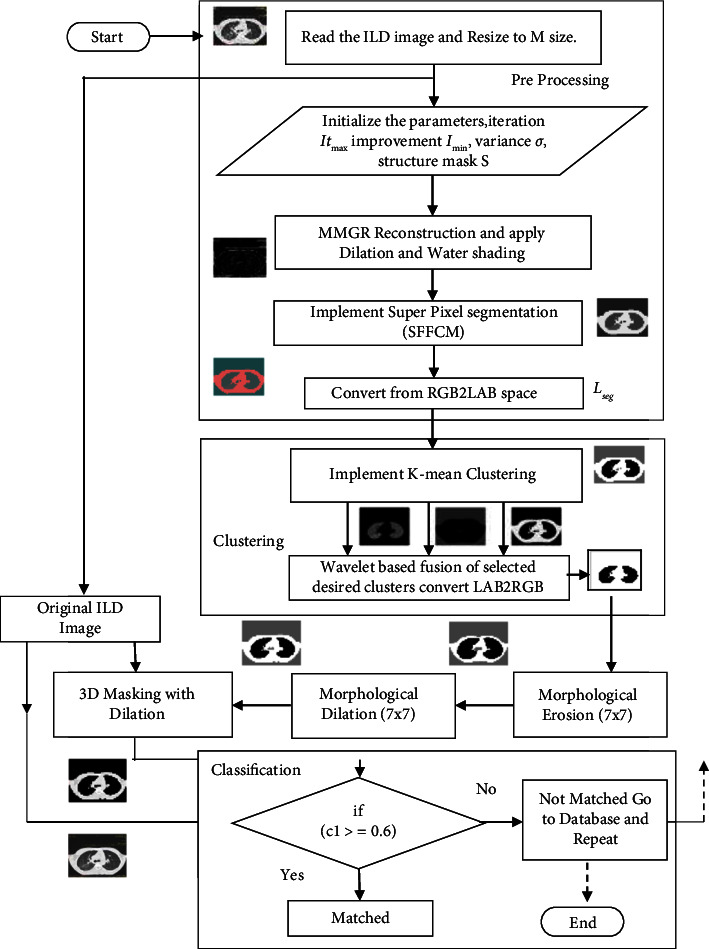
Proposed flowchart of the segmentation algorithm.

The flow chart of the proposed hybrid cluster fusion based segmentation approach is shown in Figure 11. It can be observed that a cross correlation-based template matching algorithm is used at the end for pattern classification and verification.

## 5. Experimental Setup and Performance Analysis

**Figure 12:**
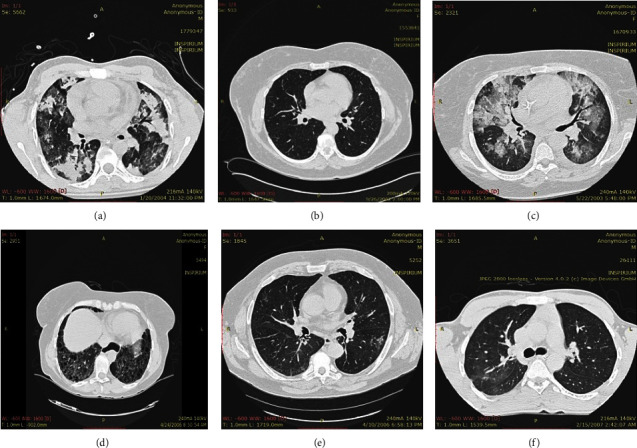
Input ILD patterns images were used for evaluation in this study. (a) ILD 1, (b) ILD 2, (c) ILD 3, (d) ILD 4, (e) ILD 5, (f) ILD 6.

The experimental results for segmentation and classification are separately presented in this paper. The test ILD images used for the quantitative evaluation of the segmentation results are shown in Figure 12.

**Figure 13:**
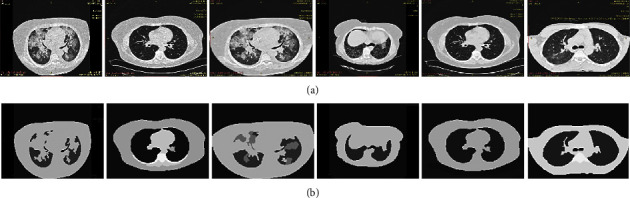
Results of the superpixel based segmentation for preprocessing stage. (a) Upper row represent the original ILD images, (b) Bottom row represents the respective super pixel images.

The first stage of the proposed algorithm is the preprocessing based on the superpixel based fast image segmentation. The qualitative results of the superpixel segmentation [39] approach are shown in Figure 13 for the ILD images. It can be observed that the main advantage of superpixel segmentation is that the backgrounds of the image are completely eliminated.

This may tend to reduce the number of classes for segregation. But a major limitation of the superpixel based segmentation method is [53] that the desired ILD patterns are not properly segmented. Some of the features are in the ILD segmented patterns. Thus, using the superpixel based method independently is not suitable for classification of the ILD patterns. Therefore, in this paper, superpixel segmentation is used as the preprocessing stage to eliminate the background.

**Figure 14:**
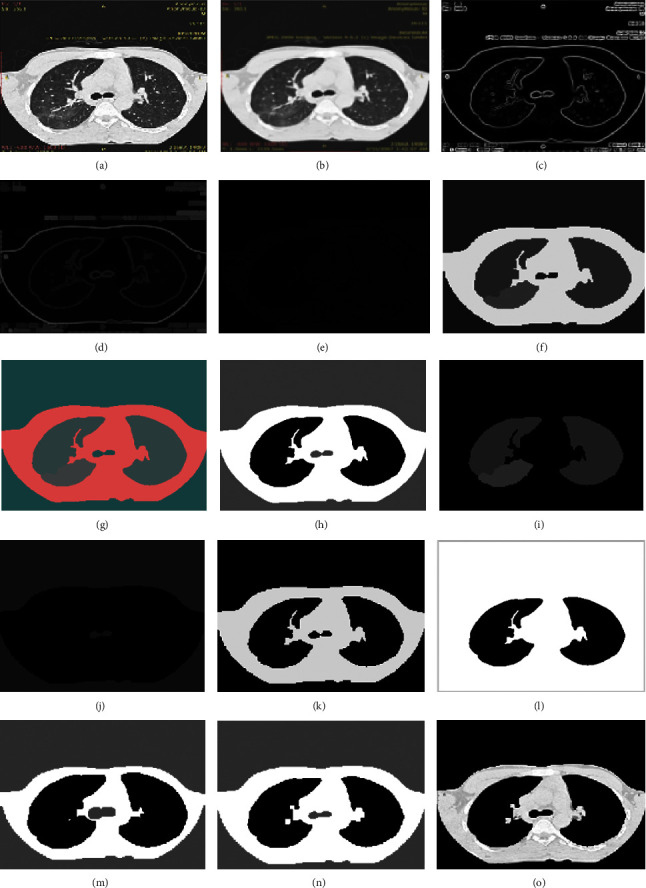
Sequential results of the proposed SPFKMC based segmentation algorithm are presented for the ILD pattern images. (a) Original image (b) Gaussian filtered (c) Gradient image (d) MMGR reconstruction (e) Watershade image (f) Super pixel segmentation (g) RGB2 LAB conversion (h) *K* mean clusters (i) *K* mean cluster 1 image (j) *K* mean cluster 2 image (k) *K* mean cluster 3 image (l) Wavelet fused cluster image (m) Erode segment image (n) Dilated image (o) Masked color segment image.

Sequential results are shown for proposed hybrid Super Pixel-based Fused K-Mean Clustering (SPFKMC) method using the Morphological masking and are shown in Figure 14. The first row of the sequential result shows the proposed method; it can be observed that the first two rows (a-f) represents the preprocessing stage results of superpixel segmentation; the Row 3 and 4 as (g-l) represent the K-mean clustering-based segmentation using wavelet fusion. The last row (m-o) represents the sequential results of the morphological masking for color segmentation.

**Figure 15:**
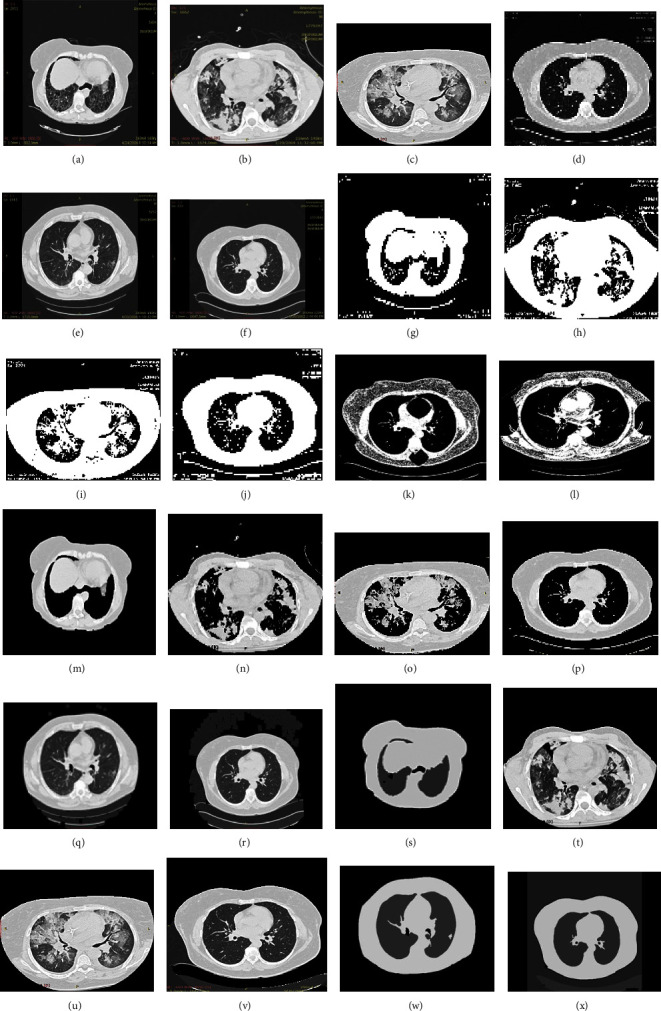
Original images with ILD patterns (a–f), output image with FCM (g–l), K-means clustering (m–r), output image with proposed method (s–x).

Figure 15, shows the ILD images with different patterns and the resultant images by K-means clustering and proposed method.

**Figure 16:**
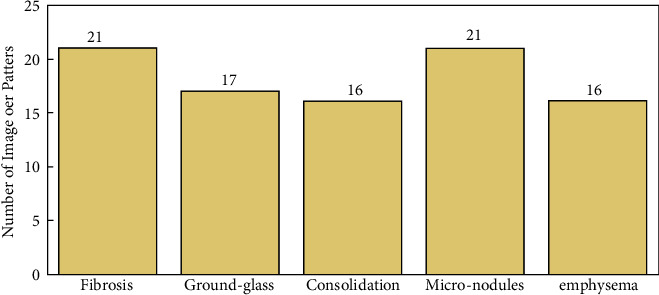
The database images are used per category of patterns for quantitative evaluation.

For the quantitative analysis and evaluation, the paper uses a publicly available ILD database for validation [73]. The library contains 128 patients influenced by one of the 13 histological completions of ILDs; 108 image plains with more than 41 clarified lung tissue designs as well as an extensive arrangement of 99 clinical parameters identified with ILDs. Out of these ILD images, as shown in Figure 16 in this paper, 21 images of fibrosis, 16 images of consolidation, 16 images emphysema, 17 images of ground-glass and 21 images of micronodules categories are considered for analysis.

Therefore, a total of 91 ILD images were considered in this dataset with five patters as healthy, consolidation, emphysema, fibrosis, ground glass, micronodules, pattern images were considered for performance evaluation. As the images are in DICOM format, we have converted the images to JPG format. The image is resized to 240 × 320. Then the image is segmented with *K*-means clustering, and the clusters 1, cluster 2 and cluster 3 are obtained. The object is found in which clusters are fused using wavelet-based fusion, after that the boundary is extracted and the output image is obtained. In the proposed method, before the *K*-means clustering, the image is a superpixel image obtained by morphological multi-scale inclination remaking with Watershed as a preprocessing stage. For quantitative evaluation, the paper compares the segmented output with the healthy image, which is considered the ground truth image. The result obtained with the proposed method removes the accurate boundaries, which is helpful for integrating adaptive neighboring information and reducing the number of different pixels in an image. Here we compare the segmented output with the ground truth images considered as the original image.

In order to evaluate the performance of our proposed method, we use accuracy, Jaccard similarity index (JSI) and Dice Coefficient Index (DCI). The Jaccard similarity index (JSI), also known as the Tanimoto coefficient, measures the overlap of two sets. It is defined as the size of the intersection of the sets divided by the size of their union. In other words,

(6) $$\begin{array}{c} SI\left(A\;,B\right)=\frac{\left|A\cap B\right|}{\left|A\cup B\right|}\text{,} \end{array}$$

where *A* (segmented output) and *B* (ground truth) are the two sets.

Like the JSI, the dice similarity coefficient (DSC) also measures set agreement. In this case, the measure is given by the formula of a set of A and B as

(7) $$\begin{array}{c} \text{DSC}\left(A,B\right)=2\frac{\left|A\text{ and }B\right|}{\left(\left|A\right|+\left|B\right|\right)}\text{.} \end{array}$$

This can be expressed in terms of the True Positive (TP), False Positive (FP), and False Negative (FN) as follows

(8) $$\begin{array}{c} Accuracy=\frac{\left(TP+TN\right)}{\left(FN+FP+TP+TN\right)}\text{,}\\ JSI=\frac{TP}{\left(FP+TP+FN\right)}\text{ ,}\\ DSC=\frac{TP}{\left(\left(FP+TP\right)+\left(TP+FN\right)\right)}\text{.} \end{array}$$

The quantitative evaluation of Algorithm 3 is performed experimentally to show results for parameters such as accuracy, JSI, and DSC and may tend to have a 5 to 10% variation in values. The values of JSI and DSC will be in the range of [0, 1] and higher values indicate better results in terms of segmentation accuracy.

Table 3 below shows the comparison of performance parameters in terms of accuracy, JSI, and DSC for different segmentation techniques.

**Figure 17:**
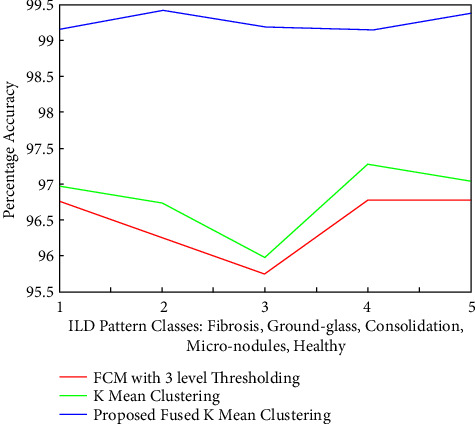
Comparisons of the percentage accuracies for five categories of ILD images segmented with FCM, *K*-means, and proposed segmentation approaches.

It can be clearly noted from Figure 17 that the average accuracy of the segmentation for the FCM approach is averaging at 96.39%, while accuracy with *K*-means has improved to an average range of 96.76%, while the proposed cluster fusion approach leads to an improved accuracy of around 99.2825%, with around 2.9% improvement over all the FCM approach. This statistical analysis justifies the quality of the segmentation results as quantitative measures. It can be observed that the accuracy is almost content for the proposed fusion approach, and this justifies the need and use of the wavelet fusion of clusters.

**Figure 18:**
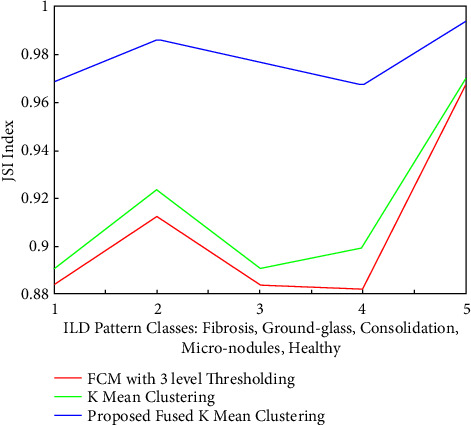
Comparisons of the JSI index for five categories of ILD patterns segmented with FCM, *K* means, and proposed segmentation approaches.

Similarly, the quantitative measures are compared in the form of a calculated JSI index shown in Figure 18. It is clear from the figure, that the average JSI for four categories of infected IDL patters is around 90.06%, 91.50%, and 97.8% respectively, for the FCM, *K*-mean and proposed fused cluster approaches. It is concluded from Figure 18 that compared to healthy images, FCM and *K*-mean clustering methods JSI index legs by around 8% and is not sufficient enough for classification. While the JSI index for the proposed fusion of the hybrid cluster approach is just less than 2% error compared to healthy images. The best JSI index is achieved for the fibrosis while the lowest index is offered for the micronodule patterns.

**Figure 19:**
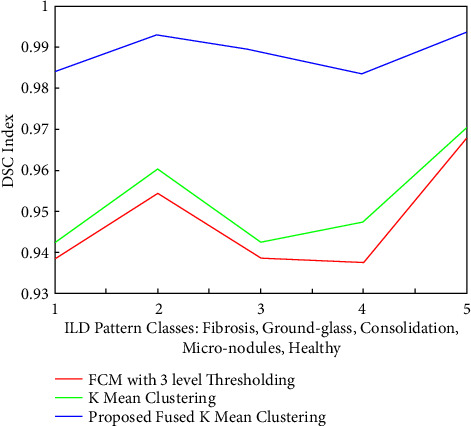
Comparisons of the DSC coefficients for five categories of ILD patterns segmented with FCM, K means, and proposed segmentation approaches.

Similarly, the quantitative measures are compared in the form of DSC coefficients shown in Figure 19. It is clear from the figure that the average DSC for four categories of infected IDL patters is around 94.212%, 94.8%, and 98.723%, respectively, for the FCM, *K* mean and proposed fused cluster approaches. It is concluded from Figure 19 that compared to healthy images, FCM and *K*-mean clustering methods DSC coefficient are off by around 2-3% error, while the proposed hybrid fusion-based segmentation approach offers the lower error rate of less than 1% compared to the healthy images case. Thus, these quantitative evaluations clearly justify the efficiency of the proposed algorithm over the existing approaches.

## 6. Results with Fusion

**Figure 20:**
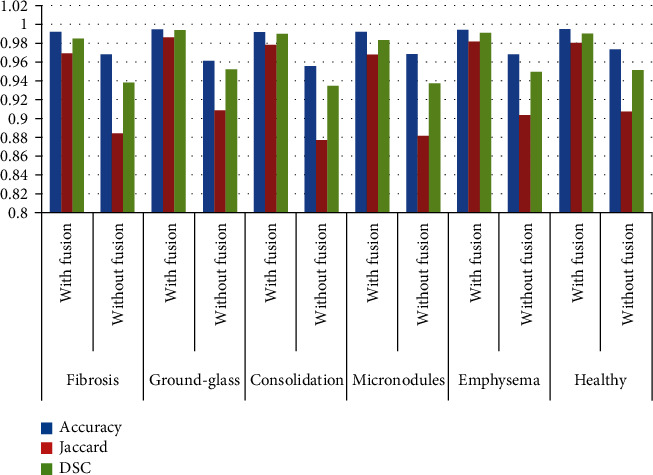
Comparison of the segmentation results with and without using the proposed wavelet fusion algorithm.

As the output obtained in cluster images by *K*-means clustering, as cluster1, cluster2 and cluster3 objects are found either in cluster1, cluster 2 or cluster 3. Therefore, it is necessary to fuse the cluster images that contain the desired output.  Figure 20 shows the results without fusion and with fusion, and it can be observed that the parameters get improved by using the proposed wavelet-based fusion.

Figure 20, *K*-means clustering results with and without fusion for different ILD patterns. It can be clearly observed that by using fusion, one may significantly improve the parametric and segmentation performance. In particular, the JSI index is significantly improved by using the fusion approach. JSI increased by more than 8% for fibrosis, ground glass, and micronodules. The accuracy is increased by around an average of 2.05% over all database images. Similarly, the DSC index average increased by around 4%. This in turn signifies the effectiveness of our proposed segmentation approach.

**Figure 21:**
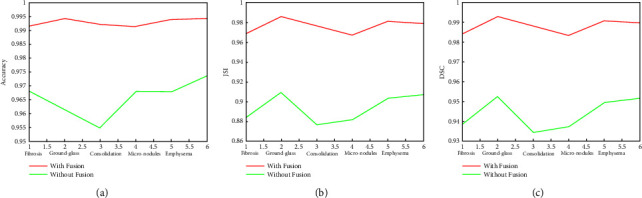
quantitative comparison of the proposed segmentation algorithm with and without fusions of clutters. (a) Accurcy comparsion plot (b) JCI plot (c) DSC comparision plot.

It is clear from the quantitative comparison presented in Figure 21 that proposed wavelet-based cluster fusion approach significantly improves the results by around 3% for the accuracy, 7-8% for JSI, and 5% for DSC for the segmented results.

## 7. Conclusion

Lung segmentation is a very crucial and important step in the detection and diagnosis of lung disease, including ILD in its prior stages. As number of research works have been given by various authors for ILD segmentation and shown their effectiveness in different cases. Proposed a hybrid approach using a superpixel-based *K*-means clustering algorithm with wavelet-based fusion and morphological operations for the segmentation. The algorithm was tested on nearly 91 images of consolidation, emphysema, fibrosis, ground glass, micronodules, pattern categories. The preprocessing stage is performed by the superpixel approach. The segmentation stage is performed by FCM, *K*-means clustering, and other proposed algorithms. As segmentation with K-means clustering need to identify the object present in either cluster and combine required cluster by wavelet-based fusion.

The results with fusion and without fusion show the fusion results are better in terms of accuracy, JSI, and DSC parameters. After that, postprocessing and classification are performed. The proposed SPFKMC algorithm (superpixel fusion based *K*-mean clustering) gives accuracy of 99.28%, DSC 98.72%, and JSI 97.87%. The results with the proposed method SPFKMC improved compared to the state-of-the art of K-means clustering and FCM for ILD image segmentation. The results actually demonstrate the superiority of the proposed SPFKMC algorithm.

### 7.1. Future Scope and Limitations

The proposed algorithm is semiatomic as it requires a user interface to identify the object present in which cluster and give the number of clusters to be fused with a wavelet-based fusion technique. In the future, the algorithm can be compared with other segmentation techniques and classification methods.

## Figures and Tables

**Algorithm 1:**
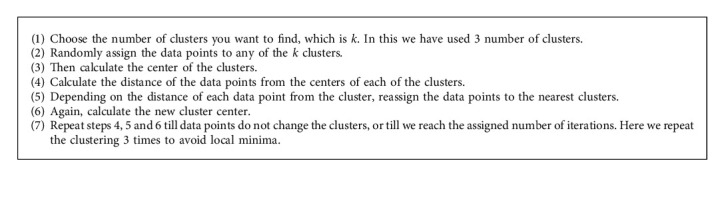
*K*-Mean segmentation

**Algorithm 2:**
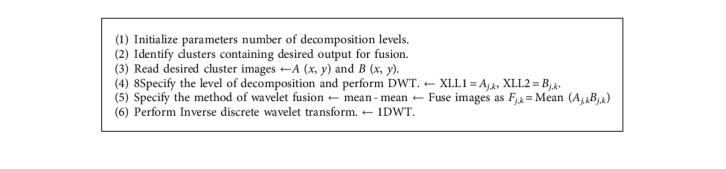
Wavelet Fusion.

**Algorithm 3:**
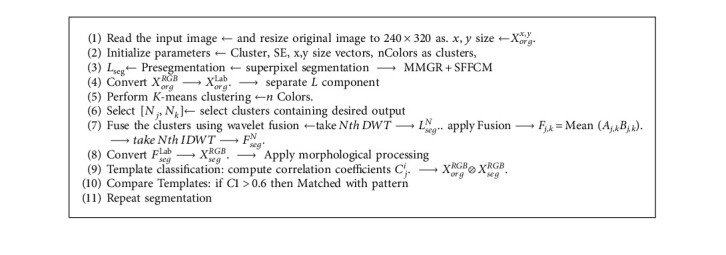
Proposed Hybrid cluster Fusion.

**Table 1:** Nomenclature used for the study.

Short form	Abbreviation
ILD	Interstitial lung disease
CT	Computer tomography
FCN	Fully convolution networks
ANN	Artificial neural network
HC	Hard clustering
ROI	Region of interest
SE	Structuring element
DWT	Discrete wavelet transform
PLM	Pixel level maxima
DSI	Dice similarity coefficient
JSI	Jaccard similarity index
SPS	Superpixel segmentation
FCM	Fuzzy *C* - means clustering
HRCT	High-resolution CT
CNN	Convolution neural network
HMRF	Hidden markov random field
SC	Soft clustering
MGR	Morphological gradient based reconstruction
IDWT	Inverse discrete wavelet transform
TP	True positive,
FP	False positive,
FN	False negative

**Table 2:** Summary of the ILD image segmentations and classification methods.

Author/Protocol	Methodology	Segmentation	Classification	Limitation/Challenges
Pang et al. [1]	Segmentation of the ILD image using deep learning of texture features	Texture based	No	Method is lengthy and needs accurate training
Saood and Hatem [2]	Deep leaning based comparison of *U*-net and SegNet for lung segmentation	Multi class segmentation	Binary classification	But method only suited for two class problem
Jalal [5]	Fuzzy based FCM for lung image segmentation and deep classification based on CNN.	Fuzzy *C* mean clustering	CNN based classification	Class imbalance ins major limitation of CNN, while FCM is sensitive to noise.
Soltani-Nabipour et al. [28]	A region growing based segmentation approach for the detection of lung tumour	Region growing	No	Simplest and depend on close pixel proximity.
Khandelwal et al. [36]	Proposed to segment the MRI images based on the 3-class FCM and thresholding-based clustering.	Thresholding and 3 class FCM	No	Method needs to optimize the threshold for better segmentation
Prajawal [44]	Various segmentation methods are classified as hard or soft methods	FCM and *K*-mean segmentation	No	Soft segmentation may not be enough to segment deep feature sets as of ILD patterns.
Gomathi Thangaraj [45]	Segmentation of the lung CT images using the probability-based FCM clustering	FCM-based segmentation	No	Method is probabilistic and the result may vary with ILD datasets
Sinaga and Yang [49]	An new approach of unsupervised *K*-mean clustering-based segmentation	*K*-Mean clustering	No	May leads to selection of the number of optimum clusters and object may belong to multiple classes
Liu et al. [53]	Lung segmentation is based on the deep random forests as a combined method of multi-scale super pixel segmentation.	Superpixel segmentation	No	But the method is unable to find the exact shape of the lung mass
Rela et al. [54]	Superpixel based segmentation using fast FCM clustering for the tumor	Superpixel segmentation	No	The number of clusters are limited
Ummay et al. [57]	Classification problem for ILD database	No	Deep CNN	Need good training knowledge
Wang et al. [65]	Superpixel based image region tracking	Superpixel	No	Used for region tracking only

**Table 3:** Comparison of 3-Class FCM and *K*-means clustering with the proposed method.

Image	Parameters	Segmentations method		
		FCM	*K*-means clustering	Proposed method
Fibrosis	Accuracy	96.76%	96.97%	**99.14%**
	JSI	0.8839	0.8907	**0.9686**
	DSC	0.9384	0.9422	**0.9841**

Ground-glass	Accuracy	96.27%	96.74%	**99.42%**
	JSI	0.9126	0.9237	**0.9862**
	DSC	0.9543	0.9603	**0.993**

Consolidation	Accuracy	95.74%	95.98%	**99.20%**
	JSI	0.884	0.8908	**0.9779**
	DSC	0.9385	0.9423	**0.9888**

Micro-nodules	Accuracy	96.77%	97.28%	**99.13%**
	JSI	0.882	0.8997	**0.9674**
	DSC	0.9373	0.9472	**0.9834**

Healthy	Accuracy	96.79%	97.04%	**99.38%**
	JSI	0.9042	0.911	**0.9813**
	DSC	0.9497	0.9534	**0.9906**

Emphysema	Accuracy	97.23%	97.48%	**99.42%**
	JSI	0.9035	0.9119	**0.9795**
	DSC	0.9493	0.9539	**0.9896**

## Data Availability

Building a reference multimedia database for interstitial lung diseases. Depeursinge A, Vargas A, Platon A, Geissbuhler A, Poletti PA, Müller H. In: Computerized Medical Imaging and Graphics, 36:3(227-238).
